# Optimizing ambulance location based on road accident data in Rwanda using machine learning algorithms

**DOI:** 10.1186/s12942-025-00400-2

**Published:** 2025-08-27

**Authors:** Gatembo Bahati, Emmanuel Masabo

**Affiliations:** 1 African Center of Excellence in Data Science (ACE-DS), College of Business and Economics, University of Rwanda, 4285, Kigali, Rwanda; 2 African Center of Excellence in the Internet of Things, University of Rwanda, 3900, Kigali, Rwanda

**Keywords:** Emergency response time, Road accident, Hotspots for ambulance location, Machine learning

## Abstract

**Background:**

The optimal placement of ambulances is critical for ensuring timely emergency medical responses, especially in regions with high accident frequencies. In Rwanda, where road accidents are a leading cause of injury and death, the strategic positioning of ambulances can significantly reduce response times and improve survival rates. The national records of Rwanda reveal a rising trend in the number of road accidents and deaths. In 2020, there were 4203 road traffic crashes throughout Rwanda with 687 deaths, data from 2021 demonstrated 8639 road traffic crashes with 655 deaths. Then in 2022 national statistics indicated 10,334 crushes with 729 deaths. The study used emergency response and road accident data collected by Rwanda Biomedical Centre in two fiscal years 2021–2022 and 2022–2023 consolidated with the administrative boundary of Rwandan sectors (shapefiles).

**Methods:**

The main objective was to optimize ambulance locations based on road accident data using machine learning algorithms. The methodology of this study used the random forest model to predict emergency response time and k-means clustering combined with linear programming to identify optimal hotspots for ambulance locations in Rwanda.

**Results:**

Random forest yields an accuracy of 94.3%, and positively classified emergency response time as 926 fast and 908 slow. K-means clustering combined with an optimization technique has grouped accident locations into two clusters and identified 58 optimal hotspots (stations) for ambulance locations in different regions of Rwanda with an average distance of 1092.773 m of ambulance station to the nearest accident location.

**Conclusion:**

Machine learning may identify hidden information that standard statistical approaches cannot, the developed model for random forest and k-means clustering combined with linear programming reveals a strong performance for optimizing ambulance location using road accident data.

## Background of the study

Accidents on the road are significant worldwide medical care concerns that contribute to an average of 1.19 million people dying globally each year. Around 650 million individuals worldwide live with impairments induced by damages from road accidents. Among these, over 90% of unintentional damages and impairments occur across countries with low and medium incomes [1]. Nearly 60% of crash mortality occurs among those aged 15 to 44, with men accounting for more than 75% [2]. From 1990 to 2023, the worldwide statistic of fatal crashes on roads has continuously increased, with the bulk of deaths happening across countries with low and medium incomes and Africa having the greatest incidence of road traffic fatalities. Statistics show that the sub-Saharan region of Africa has the highest percentage of road collisions globally, at 27 per 100,000 inhabitants [3]. The General Assembly of the United Nations and World Health Organization started a “Decade of Action for Road Safety” establishing an international strategy to address crashes road fatalities and injuries to prevent at least half of all road traffic fatalities and injuries by 2030 [4].

Rwanda is a sub-Saharan African country with 13,246,394 people based on the fifth Housing and Population Census, held in 2022. The national records of Rwanda reveal an increasing trend in road accidents and fatalities. Throughout 2020, Rwanda recorded 4203 road crashes involving 687 deaths, data from 2021 demonstrated 8639 road traffic crashes with 655 deaths. Then in 2022 national statistics indicated 10,334 crushes with 729 deaths [5]. Rwanda has a reasonably dense road network. The country’s road density is roughly 0.53 km per square kilometer of land area, ranking among the highest in the sub-region. This density improves connection across the country, making road network-based assessments very useful [6].

Several studies examined historical accident data to determine aspects contributing to road accidents, leveraged machine learning algorithms to identify road accident hotspots and used a combination of machine learning algorithms with optimization techniques to enable flexible and adaptable ambulance dispatch strategies. Variables like climate, volume of traffic, road quality, driver behavior, and accident time were commonly analyzed and identified by many researchers as the factors that contribute to road accidents.

For instance, poor lighting and adverse weather factors would make road accidents more serious. Low light factors can raise the danger of a car accident because they diminish driver sight, increase reaction time, and make it difficult to analyze road conditions. Inadequate street illumination worsens these driving hazards by making it more difficult for drivers to notice pedestrians, road signs, and potential impediments [7]. According to Wang et al. [8] findings, seasons like summer and winter are statistically connected to road accidents. Based on a study conducted in Saudi Arabia, the probability of traffic collisions increases dramatically as the ambient temperature rises throughout the summer [9]. A larger proportion of crashes happened in severe weather conditions throughout the summer (82%) than in the winter (62%) [10].

Additionally, the design and quality of the road have a significant influence on the safety of the road. Accidents can occur on roads that are poorly maintained with potholes, inadequate signs, and a lack of proper lighting. Road structure features like sharp curves, small lanes, and road intersections lacking traffic signals can increase accident risks. Road surface quality is highly connected with road traffic accidents, particularly on bend (curve) routes. They concluded that the quality of asphalt roads serves as a major outside factor causing traffic incidents. Wet asphalt roads are for cars that move extremely slowly [11].

The study conducted by Jean de Dieu et al. [12] used logistic regression (LR) and discriminant analysis to predict the contributing characteristics linked with accident severity in Rwanda. The RL findings demonstrated that all anticipated factors were statistically significant and had an effect on the number of crashes in Rwanda. The LR model was accurately identified, with 75.4% total accuracy. The findings of discriminant analysis indicated that all explanatory factors had a high and lower influence, with a significance level less than 0.05. The discriminant function was reclassified with 64.2% accuracy.

Machine learning algorithms have been widely utilized to anticipate road accident hotspots by examining past road accident data and understanding patterns. Different predictive models have been employed to gain insight into the historical road accident dataset because of their capacity to analyze complex datasets and deliver reliable predictions. LR algorithms can be employed to evaluate accident probability in specific areas. LR is a highly effective algorithm for predicting road accidents due to its cheap data needs and easy analysis structure. LR models have been utilized to evaluate accident data and identify significant contributing elements [13]. Study done by Joni et al. [14] analyzed road traffic accidents in Baghdad using the LR algorithm. Their study used binary RL with independent variables like season, weekday, and traffic defects to predict the severity of accidents, and the trained algorithm achieved 80% accuracy. According to Jean de Dieu et al. [15] in their study of making comparisons of supervised machine learning techniques in developing a forecasting model for road traffic crashes in Rwanda, they found that an RF classifier performed well with an accuracy of more than 97% in predicting accident severity.

According to Amorim et al. [16] used ANN to identify high-risk zones on federal roads of Brazil based on their possibilities of traffic crashes. A difficult road section indicates that, given a certain combination of conditions, that portion is vulnerable to major crashes. ANN achieved 83% accuracy, 84% precision, 83% recall, and an F1 score of 82%. A Random Forest (RF) mixes numerous decision trees to improve forecast accuracy while minimizing overfitting. RF works exceptionally well with big datasets and several predictive variables. RF was used to detect road accident hotspots in the interstate of Michigan. RF demonstrates an accuracy of 76.7% and 74% for validation and prediction [17]. Elsahly and Abdelfatah [18] used RF to classify road accident hotspots and realized that RF is a reliable and effective technique for discovering road incidents. Moreover, K-Means Clustering is classified under an unsupervised machine learning approach and makes categorization into k distinct clusters predicated on the similarity of their features. Study done by Holmgren et al. [19] employed a clustering algorithm to detect hazardous regions within an urban road infrastructure, and found that Clustering is powerful in identifying perceived high-risk zones for bikers in urban road networks.

Combining optimization techniques with machine learning models enables flexible and adaptable ambulance dispatch strategies. Machine learning algorithms predict accident hotspots, while optimization techniques find the optimal sites for ambulances to ensure timely responses. Optimal ambulance locations based on expected demand are achieved by optimization techniques like linear programming, mixed-integer programming, and metaheuristic approach. Riley et al. [20] applied optimization techniques with a machine learning model to predict demand zone by zone over time, and model predictive control optimization to re-balance unused trucks. They utilized column creation to serve all demands with fewer vehicles and reduced delays.

Based on the study done by Chen & Yu [21] they have used integer programming in conjunction with network-based partitioning to derive the temporary locations for the on-site EMS sites and improve EMS efficacy following the disaster. They investigated the transport infrastructures where these EMS activities take place.

The data on road accidents and deaths in Rwanda highlight the urgent need for efficient emergency medical services (EMS) to offset the escalating impact of accidents. Despite improvements in healthcare infrastructure, issues such as insufficient coverage, limited resources, and unpredictable emergency response times persist, endangering lives. To overcome these issues, this research study intends to improve ambulance placement by using machine learning algorithms to assess accident data and identify high-risk zones. By filling this gap in existing studies, this research aimed to enhance EMS efficiency, reduce response times, and ultimately save more lives. The study’s findings are expected to assist policymakers, healthcare practitioners, and emergency services, resulting in improved ambulance dispatch tactics and better outcomes for accident victims in Rwanda. By leveraging machine learning techniques with emergency response and road accident data to evaluate and identify high-risk zones, Rwanda can increase EMS efficiency, minimize response times, and save more lives.

While previous research gives useful insights into machine learning applications in ambulance optimization, there is also a lack of specific research within the context of Rwanda. As a result, the objective of this study is to utilize machine learning algorithms to optimize ambulance locations in Rwanda based on road accident data. This study intends to increase EMS efficiency, minimize response times, and ultimately save more lives by filling this important gap in the existing literature.

## Emergency medical services in Rwanda (EMS)

Rwanda’s Emergency Medical Services (EMS) also known as Service d’Aide Médicale Urgente (SAMU), was founded in 2007 by the Ministry of Health to offer prehospital emergency treatment and medical evacuation. EMS runs a nationwide dispatching centre available via the 912 hotline, which handles around 3000 monthly calls [22]. The operation centre not only dispatches emergency teams, but also offers health advice to callers who do not require ambulances. To ensure expert medical care, each ambulance deployed in Kigali is accompanied by a driver, nurse, and anesthetist. The agency oversees a fleet of ambulances scattered across the country, with 12 devoted to Kigali. The government’s goal is to have one ambulance per 20,000 people, which exceeds the World Health Organization’s requirement of one per 40,000 to 50,000 [23]. Rwanda’s hospital network includes 499 health centres, 680 health posts, 42 district hospitals, and four national referring hospitals: Kigali University of Teaching Hospital (CHUK), Butare University of Teaching Hospital, King Faisal Hospital Kigali, and the Rwanda Military Hospital. CHUK, the main referral hospital, has 519 beds, whereas King Faisal Hospital, which has been open since 1994, provides specialized treatment with 160 beds and modern amenities [24]. The EMS division continues to play an important role in addressing life-threatening crises and inter-facility transfers, and continued investments demonstrate Rwanda’s commitment to improving its healthcare infrastructure.

## Methods

The methodology used in this study involves several phases including data collection, preprocessing, performing Exploratory Data Analysis (EDA) to identify regions with high accident density, machine learning model development to predict emergency response time, model evaluation, and identification of optimal hotspots for ambulance location through the optimization process. Finding the best ambulance placements was the objective of this study to minimize emergency response times and coverage areas.

### Data description and collection

The emergency response and road accident dataset used in this study was secondary data collected by the Rwanda Biomedical Centre (RBC). Data was made up of 23,715 records of various accident interventions made by the emergency response services in Rwanda and among them, 9936 were the road accident records for the whole country of Rwanda recorded in two fiscal years 2021–2022 and 2022–2023. Also, the study used the secondary data collected by Environmental Systems Research Institute, Inc (ESRI) Rwanda to integrate the emergency response and road accident data with the Rwandan administrative sector shapefile to localize road accidents. This was done to include the geographic location of road accidents in the study. The data was useful for recognizing patterns and trends in emergency responses and road accidents. The data comprises a variety of variables related to road accidents and emergency responses, which were essential for analysis and model development are shown in the Table 1.

**Table 1:** The main variables collected and analyzed in the study

Names of variables	Names in the dataset	Types of variables
Emergency Response Time	Time Team Alerted	Dependent
	Time To Leave	
	Time Team On Scene	
	Notice To HF (Health Facility)	
	Time Team at HF	
Accident Location (Latitude and Longitude)	Event Sector and sector coordinates	Independent
Accidents Types	Event Details/Information Requested	Independent
Accident Time	Date	Independent
Accident Date	Hour	Independent
Severity of Injuries	Intervention Classification	Independent

### Data pre-processing phase

Data pre-processing was the basic preparation step of the dataset for successful analysis and modeling. This phase includes various procedures, including data cleaning, data transformation into appropriate formats, and data normalizing to ensure consistency and reliability for subsequent machine learning tasks.

#### Data cleaning

During this study, the major tasks were handling missing values, checking out-of-range data, setting time limits and timestamp validation, and formatting data. Errors during data collection or incomplete records were the major causes of missing data. 1545 records were been identified with the missing values. Statistical measurements of the available data and imputation techniques like median and mode (for numeric and categorical variables respectively) were used to impute those missing values. Moreover, error correction was also necessary to ensure data accuracy. This referred to identifying and correcting any noticeable mistakes, like out-of-range values, inconsistent categorical data, or erroneous timestamps [25]. Midnight crossing adjustments had been done by adding 1440 min to ensure better calculation across midnight for handling cases where the arrival of the team at the health facility occurs after midnight compared to the time the team was alerted. All variables related to time were transformed into minutes starting at midnight where 00:00 = 0 and 23:59 = 1439 min implying that 00:00 was taken as the lower and 23:59 as the upper time limit.

#### Data transformation

After the data was cleaned, it was transformed into formats compatible with machine learning algorithms. Categorical variables were converted into numerical representations since many machine learning models demand numerical input. The label encoding approach was used to convert categorical variables to numerical ones for better machine-learning modeling. Algorithms that can interpret ordinal relationships among categories may find label encoding more efficient as it gives each category a distinct number value [25].

#### Data normalization

Normalization was important during this study to guarantee that numerical variables make an equal contribution to the model. Normalization procedure ensured that no feature dominates over others because of its size. During normalization, the data were scaled in a specific range, typically between [0, 1] for better contribution in the modeling phase. Normalization improved machine learning models’ performance and convergence speed by assuring uniformity in the input data [25].

The following formula was used to normalize data to a range [0, 1]

$$\text{X}norm=\frac{\text{X}max-\text{X}min}{\text{X}-\text{Xmin}}$$

where: X: the original value of the feature. Xmin: denotes the lowest value of the feature. Xmax: Maximum value of the feature. Xnorm: the normalized value.

### Performing exploratory data analysis to identify regions with high accident density

EDA is an important phase in understanding a dataset since it summarizes its primary characteristics and visually represents data to reveal patterns, trends, and correlations between variables. EDA incorporates descriptive statistics and other visualization approaches to deliver insights guiding the next modeling phase [26].

#### Kernel density estimation (KDE)

KDE was used in this study to visualize the region with high accident density. It is a non-parametric estimation of the probability density function of a random variable; the KDE technique is used mainly for making out the distribution of data in a continuous space, providing some power of visualization of patterns and hence allowing the detection of regions of high or low concentration especially under spatial data analysis [27].

#### Heat maps

Heat maps utilize color to represent values from data in a matrix style, making it simple to identify a significant relationship between several variables. They are especially useful for visualizing correlation matrices, in which the color intensity represents the strength of the correlation between variable pairs [28].

### Machine learning model development to predict ambulance location

Machine learning is the subfield of artificial intelligence where machines develop the ability to learn on their own from examples given from multiple sources to extract useful insights into the data [29]. The advantage of machine learning models is the possibility of discovering unthought-of patterns, hence integrating data-driven interpretations besides those already noted by human experts [30]. To optimize ambulance locations based on road accident data in Rwanda, the K-mean clustering combined with linear programming and RF algorithms was developed.

#### K-Means clustering algorithm

K-means clustering is a very common unsupervised learning technique for classifying data points into non-overlapping clusters or groups [31]. This study used a K-means clustering approach to indicate clusters of hotspots that had road accidents based on geographical coordinates. The primary purpose was to group road accident locations to find areas with high occurrence density, which can be used to place ambulances. In addition, an important issue of K-Means Clustering was to determine the appropriate number of clusters (k) [32]. Silhouette analysis was used to select the appropriate value of k, which would minimize intra-cluster variance and maximize inter-cluster variance. Once k was chosen, K-means grouped all accident locations into k distinct clusters and indicated the potential hotspots for each cluster [31]. The placement of ambulances was influenced by accident locations in places with higher accident rates, to reduce response times and improve emergency service efficiency.

#### Silhouette analysis

The Silhouette Coefficient in K-means Clustering gives a measure of how similar a data point is within its cluster regarding cohesion to other clusters. The Silhouette analysis coefficient has values ranging from −1 to 1. This means that for 1 the data points are well-clustered, showing proper grouping, 0 indicates data points are on the boundary of clusters, showing average clustering and −1 indicates data points may be improperly clustered, implying inadequate clustering. For instance, to get started on computing the Silhouette Coefficient for a specific data point: first, determine the range for k such as 1 to 10 then plot the Silhouette analysis coefficient for every value of K [33].

The below formula is used to compute the Silhouette coefficient for any given data point:

$$S(i) = \frac{b(i) - a(i)}{{\max \{ a(i),\,\,b(i)\} }}$$

where S(i): the silhouette coefficient of the data point i; a(i): average distance between i and all the other data points in the cluster to which i belongs; b(i): average of the distance from i to all clusters of which i is not a member.

#### Random forest algorithm (RF)

RF is utilized to forecast the emergency response time of the ambulance across different accidents in various locations based on historical data and different contributing variables. It is a very strong, accurate, and popular supervised method utilized to solve regression and classification issues [34]. It works by building multiple decision trees during training and then extracting the mean prediction from each tree in regression or the mode of classes in classification. RF combines numerous regressed and trained decision trees. The RF choice is based on the majority rule [35]. The model was trained, including specifications on the number of trees (n_estimators), the maximum depth, and the minimum sample size required to divide a single node. By assessing the feature relevance scores of the RF model, we could acquire insights into which variables most significantly influenced accident occurrence and severity. Moreover, the RF contains built-in features that allow it to generate subspaces randomly on the training data to obtain high prediction accuracy [36].

### Model training and evaluation metrics

Effective model training and evaluation were essential for ensuring that the machine learning model developed during this study was robust, accurate, and applicable to new data. The process required splitting the dataset and applying a variety of evaluation metrics to comprehensively examine model performance. Generally, the dataset was grouped into two categories: the training subset and the testing subset. Usually, 80% of data is allocated to the training set, which is conventionally used for model training. The remaining 20% was used as a testing set to examine the model performance to unseen data encountered during training. This split assisted in determining how effectively the models generalized to new, previously unseen data, giving an estimate of their real-world performance [37]. The evaluation metrics like accuracy, precision, Recall, and F1-score were extracted from Table 2 to evaluate the developed model. A confusion matrix is a table pattern showing various outcomes from the prediction and results of a classification problem for visualization purposes. It is a table showing all the predicted versus actual results of the classification [38]. The confusion matrix has a major impact on this study in terms of assessing the performance of the model developed.

**Table 2:** Confusion matrix architecture table

Confusion matrix		Actual	
		Positive	Negative
Predicted	Positive	True Positive	False Positive
	Negative	False Negative	True Negative

### Identification of optimal hotspot for ambulance location through the optimization process

The process of optimization is intended to use innovative approaches to identify the best ambulance location placements.

#### Linear programming

The optimization technique relies on the objective function, which aims to decrease reaction times and maximize coverage of accident hotspots. The objective function takes into account a variety of parameters, such as geographical distance, road accident density, and emergency response time. By improving these factors, the function is intended to improve ambulance deployment efficiency and effectiveness. An objective function in Linear Programming is a linear function of two choice variables. It is that kind of linear function that is required to be maximized or minimized as per the limitations. If a and b are any constants, and x and y are two decision variables, with x > 0 and y > 0, then the objective function is Z = ax + by [39]. The geographical distribution of accidents, which indicated accident hotspots, and emergency response time were considered during the identification of optimal placement of ambulance stations to ensure that ambulances arrived at accident scenes as rapidly as possible, boosting survival and recovery rates.

#### Using the Haversine formula for optimal ambulance distance calculation

In this study, the Haversine formula is used to calculate the distance between the accident location to the nearest ambulance station. Using latitude and longitude information, the Haversine formula helps to calculate the great-circle distance between two sites on the Earth’s surface. It takes into consideration the Earth’s spherical form, making it more accurate than standard Euclidean distance in global computations [40, 41]. The haversine formula involves converting longitude and latitude to radians, computing the difference, applying the haversine formula, and multiplying by the earth’s radius (typically 6371 km). The below Haversine formula was used to evaluate that distance:

$$d = 2r \cdot \arcsin \left( {\sqrt {\sin^{2} \left( {\frac{{{\text{lat}}_{2} - {\text{lat}}_{1} }}{2}} \right) + \cos ({\text{lat}}_{1} ) \cdot \cos ({\text{lat}}_{2} ) \cdot \sin^{2} \left( {\frac{{{\text{lng}}_{2} - {\text{lng}}_{1} }}{2}} \right)} } \right)$$

where d: the distance, r: radius of the earth (6317 km), lat: Latitude and lng: Longitude.

***A correction Factor*** is sometimes known as the Wiggle Factor. The Wiggle Factor is the ratio of the actual distance traveled by road to the straight-line distance between two sites. It provides for the variations and complexities of real-world road networks as opposed to straightforward, straight-line pathways [42]. The optimal correction factor relies on the design of the road network. This correction factor can be greater in rural or hilly areas due to fewer direct routes and more difficult terrain. To estimate journey lengths more accurately, a correction factor of 1.3 to 1.5 is appropriate for the road network in Rwanda. This change guarantees that ambulance station coverage and response time calculations more precisely reflect real-world situations, resulting in more effective emergency response planning [43]. The correction factor of 1.3 has been applied to enhance the reliability of ambulance station location to adjust straight-line lengths to more accurately represent the actual route distance based on the road network factor. It has been used in the absence of digital road network data.

#### Practical implementation software

Through this study, the programming language utilized is R for statistical analysis and model development through Rstudio software. RStudio is a type of integrated development environment (IDE), for the programming language R. It is an effective tool for statistical analysis, visualization, and machine learning, making it an excellent choice for this study endeavor [44, 45].

## Results

### Identifying variables contributing to emergency response time prediction

To select variables that are associated with emergency response time, an RF importance was used to select the most important variables to forecast the target variable. The findings features are shown in the graph below.

**Fig. 1:**
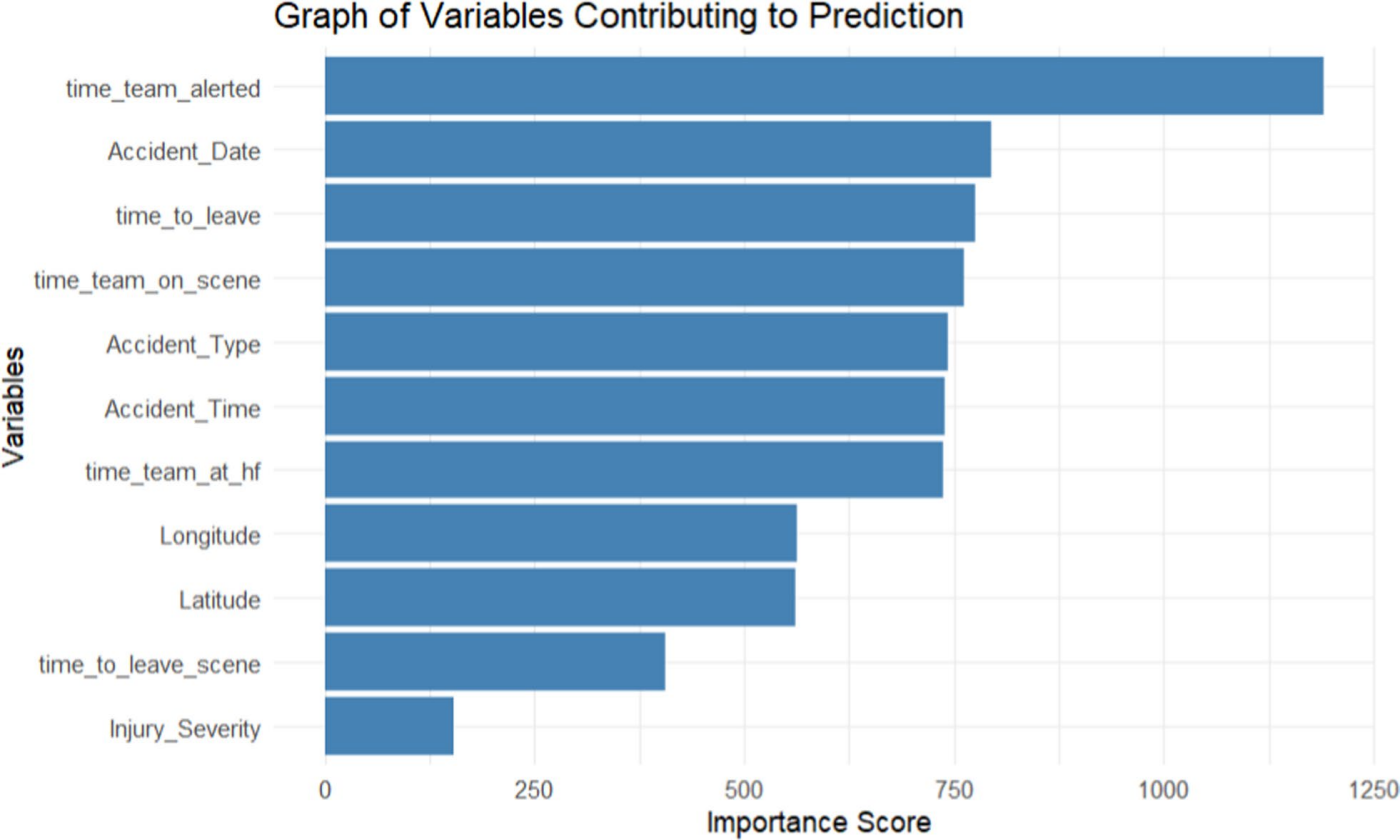
Graph of variables contributing to emergency response time prediction

The Fig. 1, shows how each feature influenced the accuracy of predicting the emergency response time. As the variable importance scores increase indicates that a feature highly influences the performance of the model in forecasting emergency response time. It displays the variables in descending order from the most influencing variable to the least variable (from top to down).

### Identification of locations with high accident density through analysis of historical accident Data in Rwanda

**Fig. 2:**
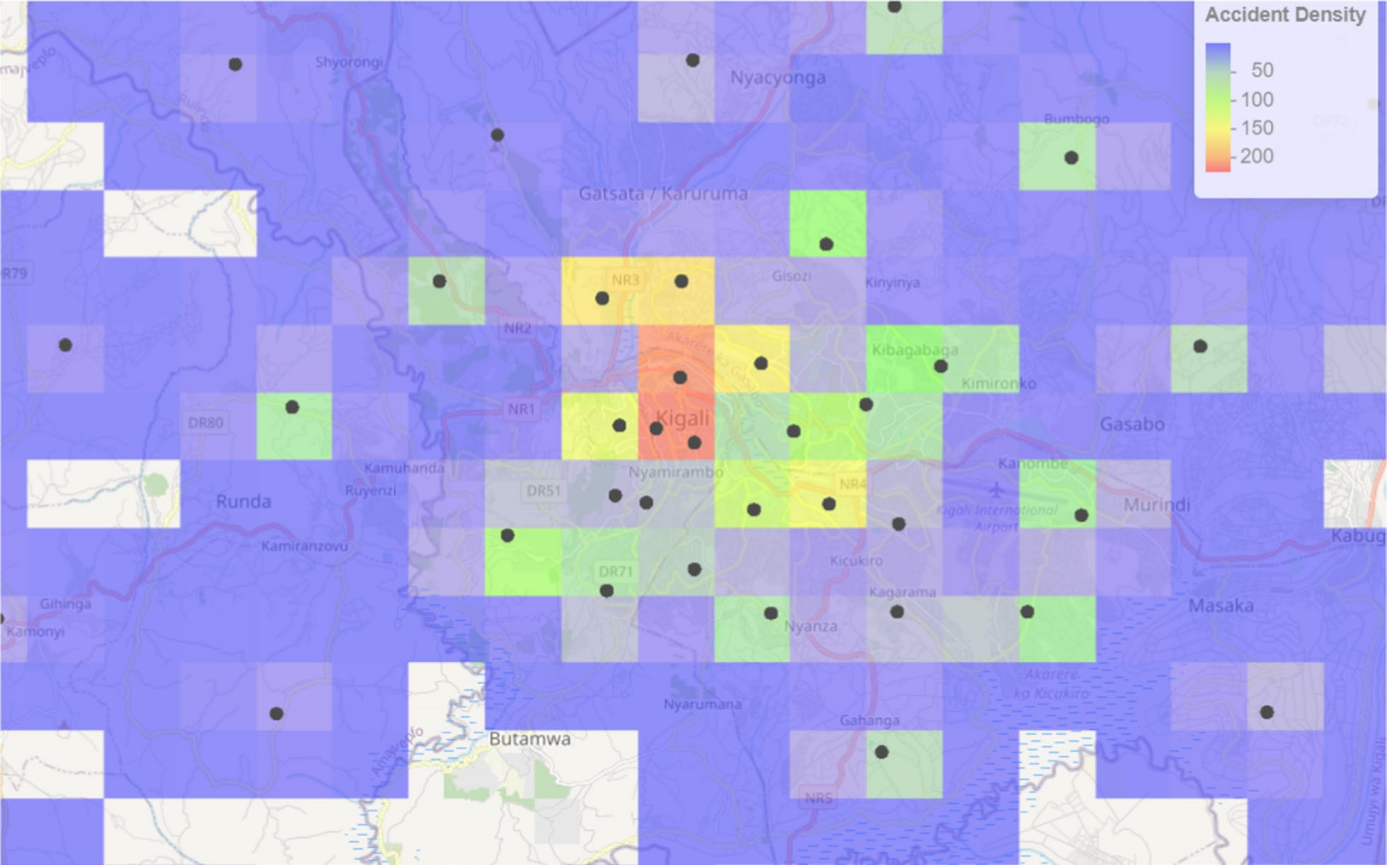
Identification of high-density regions of road accidents

Kernel Density Estimation (KDE) was used to highlight areas with high accident density indicating the areas that require more focus for accident monitoring. Kernel Density Estimation was used on the longitude and latitude of accident points with a grid size of 100*100 for the output raster. The heatmap was visualized using a leaflet map. Based on the result illustrated in Fig. 2, Kigali City and its surroundings were found to be high-risk zones for road accidents. The change in gradient color from blue/green to yellow/red shows the variation of accident densities. Blue/green regions show the regions with lower accident density while yellow/red color show the areas of high accident density. The absence of color in certain locations indicates regions with little or no accident data, which is most likely because of low incident rates or insufficient data monitoring.

### Emergency response time prediction

RF was used as a machine-learning algorithm to develop a predictive model of emergency response time with hyperparameter tuning. The RF model was evaluated on the split data where it was trained on 80% of the data and afterward tested on 20% of the data. The predictive model performance was examined with the help of evaluation metrics like accuracy, precision, recall, and F1 score in the confusion matrix. The result showed that the RF model correctly predicted emergency response time and classified it into two classes, fast and slow. It tested well the model positively predicted 926 as fast and 908 as slow. And it has negatively predicted 74 as slow and 35 as fast. And also tested well with an accuracy of 94.3%, recall of 96.3%, precision of 92.6%, and F1 score of 94.4%.

**Fig. 3:**
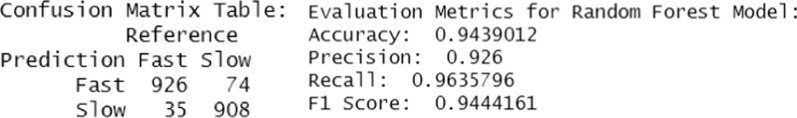
Evaluation metrics for random forest model

Figure 3 indicates that the model’s performance is good on the testing or unseen data. The 94.3% accuracy shows that the algorithm correctly forecasted the classification of response time during testing. This high accuracy indicates that the model can efficiently differentiate fast and slow reaction times according to input features. The 92.6% of the precision measure how the model correctly classifies fast instances out of all cases categorized as fast. This demonstrates the strong ability of the model to avoid false positives even though there is a small case of a slow being classified as fast.

In addition, the 96.3% recall indicates a proportion of accurately predicted fast cases among all actual fast instances in the data. This high recall reflects the model’s concentration on identifying the majority of fast responses, which is important in emergencies when quicker responses are prioritized. The 94.4 F1 Score shows the harmonic average of precision and recall, balancing both measurements. Its high score indicates a better balance between recall and precision. The F1 score demonstrates that the capacity of the model to accurately classify fast and slow replies is constant and well-rounded, making it reliable for decision-making.

Furthermore, the confusion matrix thoroughly describes how the prediction of the model relates to the actual classifications. 926 Fast cases are accurately classified as Fast (True Positives); the model accurately predicted 926 instances when the response time was fast. 908 slow cases were accurately classified as slow (True Negatives); the model accurately predicted 908 instances where the response time was correctly slow. 74 Slow cases were misclassified as Fast (False Positives); the model falsely classified 74 instances as fast response that were actually slow response time. These false positives might result in an unnecessary allocation of ambulance resources to places where quick responses are not required. However, the model’s low frequency of false positives indicates that it is not excessively aggressive in predicting fast responses. 35 fast cases were misclassified as slow (False Negatives); the model falsely classified 35 instances as slow response that were actually fast response time. These false negatives are essential because they represent missed occasions in which emergency services may not respond as soon as possible, which may help in optimizing ambulance resources for better responding.

### Identification of optimal hotspots for ambulance location in Rwanda

To identify the optimal hotspot for ambulance location, a k-means clustering algorithm was developed, combined with a linear programming algorithm.

**Fig. 4:**
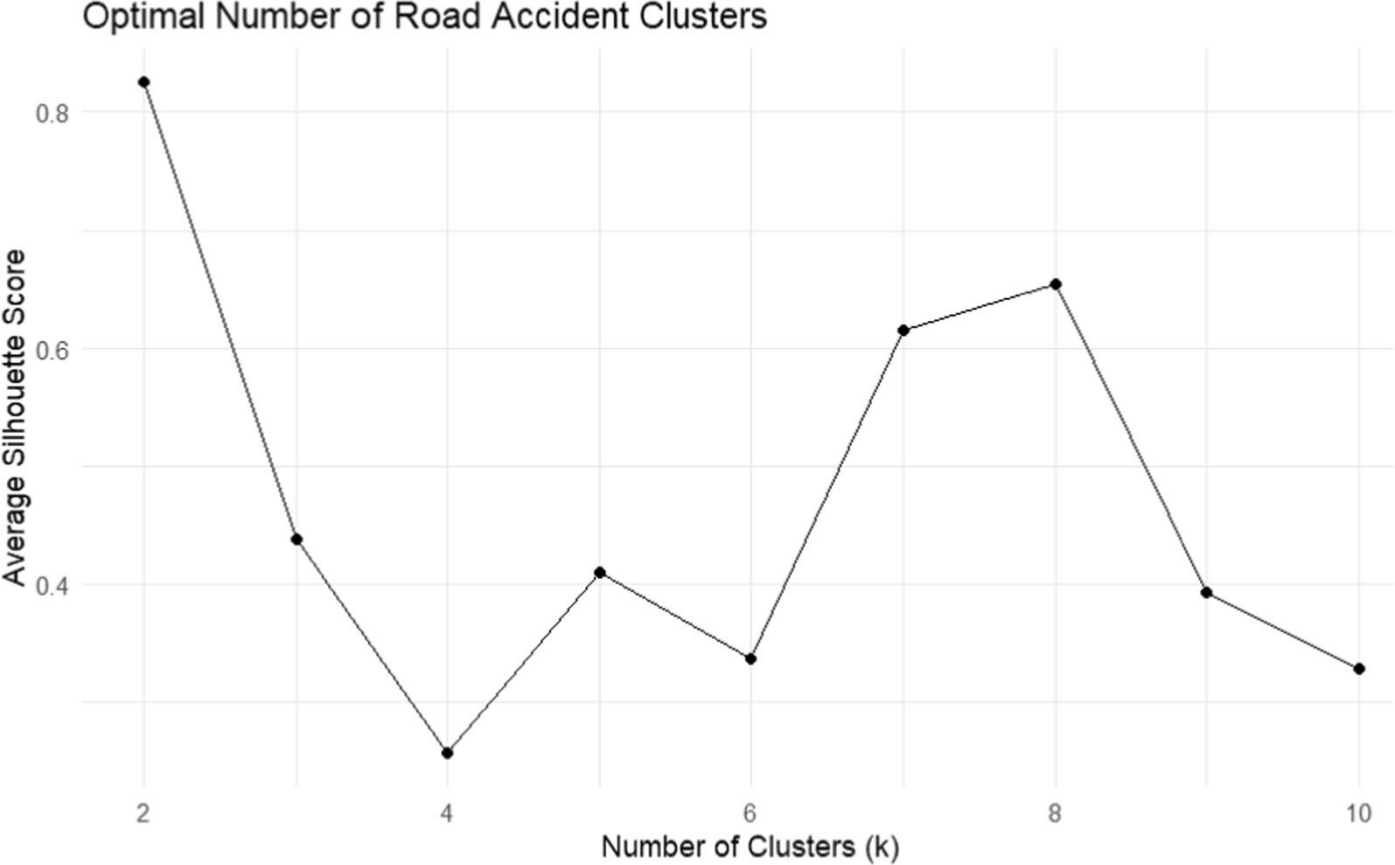
Determination of the optimal number of road accident clusters

A k-means clustering algorithm was used to divide accident locations into discrete clusters dependent on their physical closeness. Figure 4 demonstrates the optimal number of clusters, determined via silhouette analysis, which resulted in 2 optimal numbers of clusters with an average Silhouette score of 0.85. The outputs of clustering helped to group accident locations into two groups and made it easier to localize ambulance stations in each cluster.

### Linear programming formulation

In the optimization process of ambulance location, a linear programming approach was developed to reduce emergency response times while maximizing coverage of accident hotspots. The goal was to localize ambulances in a way that would reduce the maximum response time for any accident.

The calculation of ambulance hotspots (stations) was done depending on two factors:

- *Calculation of percentage-based*: Calculates the optimal number of clusters as a percentage of total accident data.
- *Minimum stations per cluster*: making sure that each cluster has enough coverage, by setting a minimum number of stations per cluster. This minimum number is equal to the number of optimal k which is 2.

The following constraints were developed for an objective function to formulate the optimization problem:

- *Objective function*: was set to improve the coverage area by minimizing the number of stations
- *Constraint*: was set to minimize response time by considering the minimum number of stations by cluster.

The optimization process of linear programming identified 58 optimal ambulance stations across the 2 clusters. These 58 stations are strategically located to cover the indicated accident clusters.

**Fig. 5:**
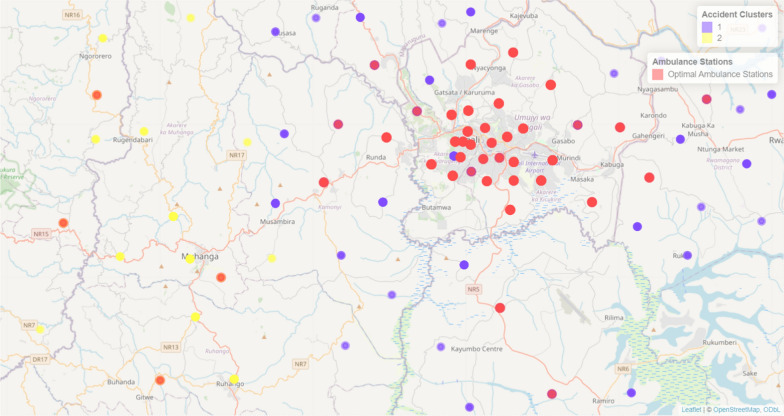
Ambulance locations hotspots (stations)

The mean distance from the locations of the accident to the closest ambulance hotspot (station) has been calculated using the Haversine formula applied on the correction factor of 1.3 and the average distance of accident locations to the nearest ambulance station is 1092.773 m. This metric represents the geographical efficiency of ambulance locations by minimizing the distance between accident locations and ambulance stations, the model tries to minimize the emergency response time. Figure 5, illustrates the identification of ambulance hotspot (stations) locations in red dots and violet and yellow dots showing the 2 clusters of road accidents.

## Discussions

This study demonstrates the application of the machine learning algorithm in the prediction of emergency response time and the identification of the optimal hotspots for ambulance location (station) based on road accident data in Rwanda, which helped improve emergency response time and services and maximize the coverage of the accident areas. The study was carried out on road accident data with 9936 records for the fiscal years 2021–2022 and 2022–2023 consolidated with Rwanda administrative sector coordinates (shapefile) data for identification of road accident locations.

A kernel density estimation was used to identify the regions with high accident density, which indicates the locations with a high rate of need for ambulance location (stations) for easier response to the accident. The variation of the color found in Fig. 2, from Blue/green regions, shows the regions with lower accident density, while yellow/red color shows the areas of high accident density. These high-density zones (hotspots) are crucial for emergency response planning because they play a role in identifying areas that may require more resources or preventative actions. The results line up with the study done by Rahman [46] to study the geographical patterns of road accident fatalities in London, the United Kingdom, to classify hotspots of road accidents.

Two machine learning algorithms were used in this study. An RF model was developed to predict the emergency response time of the ambulance and the K-means clustering algorithm combined with a linear programming algorithm was utilized to identify the optimal ambulance location (station). An RF model has predicted well the emergency response time with an accuracy of 94.3%. RF has classified emergency response time into two classes, fast and slow. It was positively predicted 926 as fast and 908 as slow and negatively predicted 74 as slow and 35 as fast. These slow classes indicate where the optimization of ambulance placement should be focused for better utilization of the resources, and also indicate the place where the current ambulance distribution was inadequate. The decision maker should prioritize the placement of the ambulance station in the region where the prediction of response time was slow for effectively minimizing response delays. And also tested well with an accuracy of 94.3%, recall of 96.3%, precision of 92.6%, and F1 score of 94.4%. The accuracy of 94.3% implies that the performance of the model is reliable in classifying emergency response time to unseen data. These findings line up with previous studies done by Ramesh Kumar and Sabiha [47] in the application of predicting emergency evacuation time where the RF algorithm was found to be useful in hazardous management.

A K-means clustering algorithm combined with a linear programming algorithm has been used to identify the optimal ambulance location (station). The k-means clustering has grouped the accidents into 2 optimal numbers of clusters with a Silhouette score of 0.85 using Silhouette analysis. The objective function has been formulated by establishing the constraints that each cluster must have 2 (optimal k) minimum number of stations and all accident clusters to be covered to minimize response time and maximize the coverage of the areas. 58 optimal ambulance stations have been identified and determined to be the optimal hotspots for ambulance stations with an average distance from accident locations to the nearest ambulance station of 1092.773 m. It implies that small, properly distributed ambulances can cover a broad range of accident hotspots, thus minimizing both operational costs and response times. The results line up with the study conducted by Dubey [48] looking for a way to position ambulances in an optimal way for road accidents. Also, the study was done by Yunus and Abdulkarim [49] on the placement of the ambulance in the optimal stations to better serve the emergency calls lined up with this.

According to [49], highlighted that the placement of the ambulance location must be selected based on various factors like accident density, emergency response demand nature and state of the city, density, and distribution of the population… which are similar to the suggestion of our study that the ambulance location should be determined based on the high demand area and accident density of region after leveraging accident data to reduce emergency response time and save life.

Based on the results and the previous studies, machine learning algorithms combined with the optimization process are powerful enough to predict emergency response time and optimize ambulance location to reduce response time and save lives.

## Conclusion

The main objective of this study was to develop a predictive model for emergency response and road accident data with the help of machine learning to optimize ambulance location. An RF model was used to predict emergency response time with an accuracy of 94.3% and classified into two classes: fast and slow. With the k-means clustering algorithm, two optimal clusters of accident locations were found using Silhouette analysis with a silhouette score of 0.85, and by applying linear programming, the model identified 58 optimal ambulance hotspots (stations) and the average distance from accident locations to the nearest ambulance station: 1092.773 m. Based on the result from the RF and k-means clustering combined with linear programming indicates the need to optimize ambulance placement. The results provide informed decisions that have helped to make sure that the emergency resources are dispatched where they are mostly needed to save lives.

This study adds a contribution to the existing knowledge by utilizing machine learning for emergency response and road accident data prediction. Future studies may try to evaluate different machine learning algorithms combined with various optimization techniques on emergency response and road accident data for better saving accident victims and reducing emergency response time.

## Figures and Tables

**Fig. 6:**
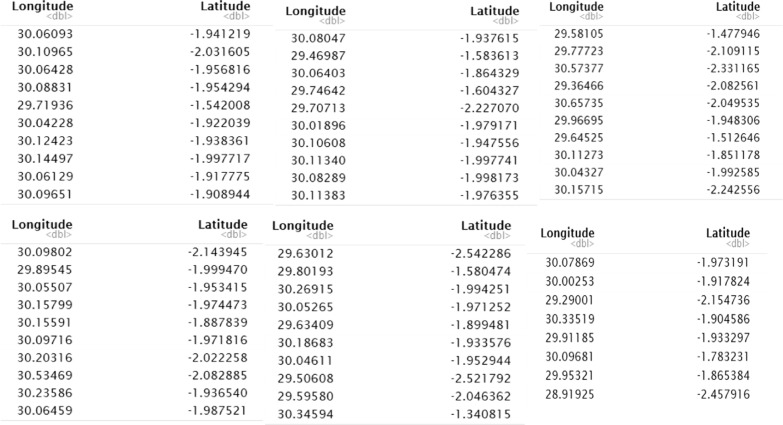
The 58 optimal ambulance locations coordinates

## Data Availability

The data that support the findings of this study are available from Rwanda Biomedical Centre (RBC) and ESRI Rwanda but restrictions apply to the availability of these data, which were used under license for the current study, and so are not publicly available. Data are however available from the authors upon reasonable request and with permission of Rwanda Biomedical Centre (RBC)and ESRI Rwanda.

## Appendix

The Table 3: Displays the variables in descending order from the most influencing variable to the least influencing variable (from top to down).

**Table 3:** Displays the variables in descending order from the most influencing variable to the least influencing variable (from top to down)

Rank	Variable	Metadata (data type and format)	Description
1	Time_Team_Alerted	Timestamp (HH:MM:SS)	The feature with the highest importance scores most contributing to the emergency response time prediction is the time the team is alerted with 1190.9273. This indicates the time the emergency team is communicated. The earlier notifications allow for a faster reaction, but delayed alerts increase the period between the accident and the arrival of the team
2	Accident_Date	Date (YYYY-MM-DD)	Accident_Date score 795.3168, is crucial in forecasting the emergency response time. The score indicates that certain dates have more difficult conditions for emergency response
3	Time_to_Leave	Timestamp (HH:MM:SS)	Time_to_leave with an importance score of 776.3370 reflects how long it took for the emergency team to get ready and leave for the accident scene after being informed. A high significance score implied that delays in departing the base station can have a major influence on response times. Strategically setting up ambulances in highly demanded locations may improve response times
4	Time_Team_on_Scene	Timestamp (HH:MM:SS)	Time_team_on_scene with an importance score of 762.1619 is the time used by the emergency response team to arrive at the scene. The high score highlighted the significance of travel time caused by different factors. This can be reduced by improving ambulance station placement and routing to allow quicker ambulance response
5	Accident_Type	Categorical	Accident_Type with scores of 742.9029, various accident types could create environmental challenges to the emergency response teams which may affect the response time. Thus, identifying the accident type allowed the model to predict more accurately the urgency encountered by emergency teams
6	Accident_Time	Time (HH:MM:SS)	Accident_Time with an importance score of 739.3785: The time of day at which the accident happened has a major influence on prediction. Response time might vary significantly for different reasons for instance during rush hour, accidents occur at night, or on weekend days/night. The high importance score indicates that the model was capable of capturing time-dependent changes in response time
7	Time_Team_at_HF (Health Facility)	Timestamp (HH:MM:SS)	Time_team_at_HF (Health Facility) with an importance score of 737.9304. this indicates the time the emergency team took to transfer the accident victim from the scene to the health facility. Its high score indicates how the variable was important in predicting the emergency response time
8	Longitude and Latitude	Numeric (Decimal Degrees)	Latitude with a score of 561.4347 and Longitude with a score of 562.5474. These 2 variables are the geographical location of the accident. Their score revealed the value of how geography played an important role in predicting emergency response times. Latitude and longitude enabled the model to account for differences in location in emergency response, emphasizing the need for ambulance station placement
9	Time_to_Leave_Scene	Timestamp (HH:MM:SS)	Time_to_leave_scene with a lower importance score of 405.9966, measure the time taken by the emergency team at the accident scene. Although it contributed to the entire response process, once the team arrived at the site, their first focus was usually on stabilizing the patient. The scene time varies less than travel-related timings such as leaving the station or arriving at the hospital
10	Injury_Severity	Categorical	Injury_Severity with the lowest importance score of 153.5285 shows its least contribution to emergency response time prediction. Emergency response time cannot directly be influenced by the injury severity but this may influence in prioritizing accident victims’ treatment

Figure 6 shows Rwanda’s 58 optimal ambulance location (station) coordinates (latitude and longitude). These coordinates indicate where ambulances should be located or stationed to ensure quick response times. They are visualized using a leaflet map, allowing for simple geographic identification of ambulance station locations around the region.

## Abbreviations

EMS: Emergency medical services
ANN: Artificial neural network
LR: Logistic regression
RF: Random forest
SVM: Support vector machine
EDA: Exploratory data analysis
